# Signature and Prediction of Perigastric Lymph Node Metastasis in Patients with Gastric Cancer and Total Gastrectomy: Is Total Gastrectomy Always Necessary?

**DOI:** 10.3390/cancers14143409

**Published:** 2022-07-13

**Authors:** Chun-Dong Zhang, Hiroharu Yamashita, Yasuhiro Okumura, Koichi Yagi, Susumu Aikou, Yasuyuki Seto

**Affiliations:** 1 Department of Gastrointestinal Surgery, Graduate School of Medicine, The University of Tokyo, Tokyo 113-8655, Japan; zhang-chundong093@g.ecc.u-tokyo.ac.jp (C.-D.Z.); yamashita.hiroharu@nihon-u.ac.jp (H.Y.); okumura-tky@umin.ac.jp (Y.O.); yagik-tky@umin.ac.jp (K.Y.); aikous-tky@umin.ac.jp (S.A.); 2 Department of Digestive Surgery, Nihon University School of Medicine, Tokyo 101-8309, Japan

**Keywords:** gastric cancer, metastasis signature, perigastric lymph node, proximal gastrectomy, pylorus-preserving gastrectomy

## Abstract

**Simple Summary:**

The signature and prediction of perigastric lymph node metastasis (pLNM) is clinically important, but evidence is still lacking. Here, we aimed to identify an informative signature for the prediction of pLNMs in gastric cancer patients after total gastrectomy, and reassess the current indications for proximal gastrectomy and pylorus-preserving gastrectomy (PPG). We found that proximal gastrectomy may be expanded to patients with stage T1–T2 GC and/or tumor diameter < 4 cm in the upper-third stomach, while PPG may be expanded to include T1–T2/N0 and/or tumors < 4 cm in the middle-third stomach. Furthermore, we developed a new predictive factor, the shortest distance from the pylorus ring to the distal edge of the tumor, which showed good predictive performance for pLNMs.

**Abstract:**

Background: A growing number of studies suggest that the current indications for partial gastrectomy, including proximal gastrectomy and pylorus-preserving gastrectomy (PPG), may be expanded, but evidence is still lacking. Methods: We retrospectively analyzed 300 patients with gastric cancer (GC) who underwent total gastrectomy. We analyzed the incidence of pLNMs in relation to tumor location, tumor size and T stage. We further identified predictive factors for perigastric lymph node metastasis (pLNM) in stations 1, 2, 3, 4sa, 4sb, 4d, 5, and 6. Results: No patients with upper-third T1–T2 stage GC had pLNMs in stations 4sa, 4sb, 4d, 5, or 6, but 3.8% of patients with stage T3 had 4d pLNM. No patients with upper-third GC < 4 cm in diameter had pLNMs in 2, 4sa, 4d, 5, or 6, and 2.3% of patients had pLNMs in 4sb. For middle-third GCs, 2.9% of patients with T1 stage had pLNMs in 4sa and 5, but no patients with T2 stage or tumors < 4 cm had pLNMs in 2, 4sa, or 5. The shortest distance from pylorus ring to distal edge of tumor (sDPD) was a new predictive factor for pLNMs in 2, 4d, 5, and 6. Conclusions: Proximal gastrectomy may be expanded to patients with stage T1–T2 GC and/or tumor diameter < 4 cm in the upper-third stomach, whereas PPG may be expanded to include T1–T2/N0 and/or tumors < 4 cm in the middle-third stomach. A new predictive factor, sDPD, showed good predictive performance for pLNMs, especially in stations 4d, 5, and 6.

## 1. Introduction

Chemotherapy is widely applied in patients with gastric cancer (GC) [1], but many patients show drug resistance. Human epidermal growth factor receptor 2 (HER2)-targeted therapy is currently gaining importance worldwide [2]. In addition, the tumor microenvironment has recently emerged as a notable therapeutic target in GC, in relation to its important role in cancer progression and drug resistance [3]. However, surgery remains the highest priority for patients with curable GC [4]. 

Adequate lymphadenectomy to remove all metastatic lymph nodes is necessary to ensure the best prognosis for patients with curable GC. A better understanding of the signature and prediction of perigastric lymph node metastasis (pLNM) is clinically important to allow the selection of the most appropriate surgical procedures, such as the type of gastrectomy and the extent of lymphadenectomy. However, the precise preoperative prediction of station-specific LNMs remains a challenge. Various procedures, including computed tomography (CT) [5], ultrasonography, magnetic resonance imaging with ferumoxtran-10 [6], and use of DNA methylation markers [7], have been explored to predict pLNMs prior to surgery; however, these have been inaccurate or complex, or have been unable to predict station-specific LNM [8,9,10]. Furthermore, the Maruyama Computer Program (MCP) was applied to predict LNMs in GC patients treated with neoadjuvant chemotherapy followed by gastrectomy [11]. However, the LNMs predicted by MCP were not station-specific and further investigations are therefore required.

The indication for partial gastrectomy is determined by multiple factors, including tumor location, histology, tumor size, sufficient resection margin, the Union for International Cancer Control (UICC) T stage, and the status of pLNMs [12,13]. For example, proximal gastrectomy is only indicated for a limited number of proximal early GCs (cT1N0) when more than half of the distal stomach can be preserved and when no lymphadenectomy is required for lymph node stations 4d, 5, and 6, according to the 5th Japanese Gastric Cancer Treatment Guidelines (5th JGCTG) [12]. However, the pLNM rates in stations 4d, 5, and 6 are similarly low in T2 or even T3 proximal tumors [14,15], indicating the possibility of expanding proximal gastrectomy to these cases. Pylorus-preserving gastrectomy (PPG) has been suggested as an alternative to distal gastrectomy for patients with early GC (cT1N0) in the middle-third stomach, with comparable oncological safety and better postoperative nutritional status and quality of life compared with distal gastrectomy [12,16,17,18,19]. However, the current indications for both proximal gastrectomy and PPG based on classic guidelines are narrow and warrant reevaluating [14,18].

The above factors highlight the need to develop an informative signature of pLNMs in GC, in order to reassess the current indications for gastrectomy with lymphadenectomy. Here, we aimed to identify an informative signature and prediction of pLNMs in stations 1, 2, 3, 4sa, 4sb, 4d, 5, and 6 in patients with GC undergoing total gastrectomy. We also aimed to reevaluate several clinical issues, including whether total gastrectomy is always necessary, or might be replaced by proximal gastrectomy for GCs in the upper-third stomach; if the current extent of lymphadenectomy for PPG is suitable; and if distal gastrectomy might be replaced by PPG for GC in the middle-third stomach.

## 2. Materials and Methods

### 2.1. Patients and Eligibility Criteria

We retrospectively collected clinical data for patients with GC who underwent total gastrectomy at the Department of Gastrointestinal Surgery, the University of Tokyo Hospital, from January 2000 to December 2013. The eligibility criteria included total gastrectomy with adequate lymphadenectomy (D1/D1+/D2), no preoperative treatment, sufficient data on the main outcomes, primary and single tumor in the stomach, no residual microscopic tumors (R0 resection), no distant metastasis (M0) at the time of surgery, and age between 18 and 80 years. We excluded patients with inadequate lymphadenectomy (less than D1), R1 or R2 resection, insufficient data on the main outcomes (e.g., clear images of the whole stomach), and patients with neoadjuvant chemoradiotherapy or chemotherapy.

Patients were followed-up routinely every 3–6 months until the last follow-up or death, and the median follow-up period was 61.4 months (range, 1–187 months). This study was reported in accordance with the Strengthening the Reporting of Observational Studies in Epidemiology (STROBE) reporting guidelines [20]. This study was approved by the ethics committees of the University of Tokyo (No. 3962).

### 2.2. Lymph Node Dissection for Total Gastrectomy

The extent of lymphadenectomy was classified as D1, D1+, and D2, as described previously [12,21]. For total gastrectomy, D1 lymphadenectomy included stations 1–7, D1+ lymphadenectomy included D1 plus stations 8a, 9, and 11p, and D2 lymphadenectomy included D1 plus stations 8a, 9, 11p, 11d, and 12a. The primary outcome was the signature and prediction of pLNMs in stations 1, 2, 3, 4sa, 4sb, 4d, 5, and 6.

### 2.3. Clinical and Pathological Factors

We reviewed the following clinical and pathological factors: sex, age, tumor size, cross section, UICC 8th T stage (T stage), UICC 8th N stage (N stage), pathological type, number of retrieved lymph nodes and positive lymph nodes in stations 1, 2, 3, 4sa, 4sb, 4d, 5, and 6, status of lymphatic invasion, status of venous invasion, and adjuvant chemotherapy. The distance from the pylorus ring to the distal edge of the tumor was measured, and the shortest distance was identified (sDPD).

### 2.4. Statistical Analysis

The pLNM rates were first analyzed in relation to tumor location. We then analyzed the numbers of patients with pLNMs in relation to the combinations of tumor location with T stage, tumor size, and lymphatic invasion. Univariate and multivariate analyses were performed using logistic regression. To identify independent predictive factors for lymph node metastasis, variables with statistical significance in univariate analysis were applied in multivariate analysis (1st). To identify independent predictive factors for pLNMs (stations 1, 2, 3, 4sa, 4sb, 4d, 5, and 6), variables with statistical significance in multivariate analysis (1st), plus two factors of interest, tumor size and sDPD, were included in multivariate analysis (2nd). The predictive performance of the factors and models were further assessed. A higher area under the receiver operating characteristic curve indicated better model discrimination, while a lower Akaike information criterion value indicated superior model fitting, as described previously [22].

Statistical analyses were conducted using IBM SPSS Statistics version 22.0 (Armonk, New York, NY, United States), R version 4.1.0 (R Foundation for Statistical Computing, Vienna, Austria), and MedCalc version 20.104 (MedCalc Software, Ostend, Belgium). A *p* value < 0.05 was defined as statistically significant.

## 3. Results

### 3.1. Patient Characteristics

**Figure 1:**
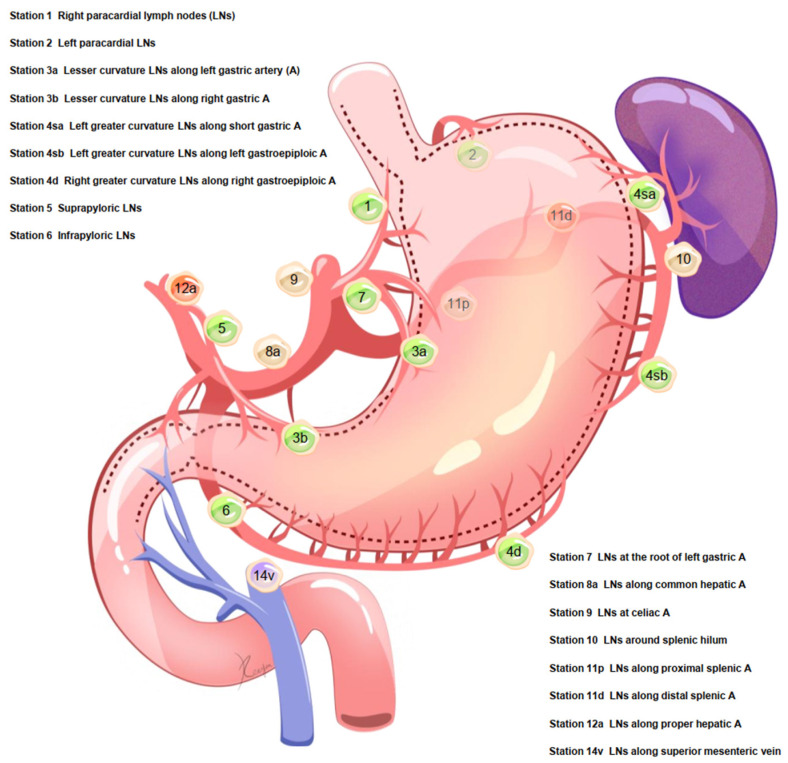
Details of lymph node stations.

Between January 2000 and December 2013, 300 GC patients who underwent total gastrectomy were eligible, including 234 (78.0%) males and 66 (22.0%) females (Table S1; Figure S1). The tumor location was classified as being in the upper- (58.7%), middle- (32.3%), or lower-third (9.0%) of the stomach. A total of 34.0% of patients were T1 stage and 56.7% were node-positive (N1–N3). The median number of retrieved lymph nodes was 48 (range, 11–132), and 98.0% (294/300) of patients had an adequate number (n ≥ 16) of retrieved lymph nodes. The incidences of lymphatic invasion (+) and venous invasion (+) were 58.3% and 68.0%, respectively. Adjuvant chemotherapy was applied in 48.3% (145/300) of patients. The details of lymph node stations 1–14v are shown in Figure 1.

### 3.2. Tumor Location May Determine pLNM Risk

The total number of retrieved lymph nodes, metastatic lymph nodes, and pLNM rates were analyzed in relation to tumor location (Table S2). Among the 176 patients with upper-third tumors, the highest pLNM rates were found in stations 3 (11.3%), 1 (10.6%), and 2 (10.2%), and the lowest rates in 6 (0%), 5 (0.8%), and 4d (1.4%). Among the 97 patients with middle-third tumors, the highest pLNM rates were observed in stations 3 (19.3%) and 4sa (18.5%), and the lowest rates in 2 (6.2%) and 6 (8.8%). For the 27 patients with lower-third tumors, the highest pLNM rates were noted in stations 3 (18.7%) and 5 (13.3%), and the lowest in station 2 (2.9%).

### 3.3. Tumor Location Combined with Tumor Size May Determine pLNM Risk

Among patients with tumors in the upper-third stomach, patients with tumors < 4 cm had no pLNMs in stations 2, 4sa, 4d, 5, or 6 and 2.3% of patients had 4sb pLNMs, while patients with tumors ≥ 4 cm had no pLNMs in station 6, and only 0.8% of patients had station 5 pLNM (Table 1; Figure S2a,b). Among patients with middle-third tumors, patients with tumors < 4 cm had no pLNMs in stations 2, 4sa, 4sb, 5, or 6 and 4.3% of patients had station 1 pLNM, while the incidences of pLNMs in patients with tumors ≥ 4 cm ranged from 6.8% (station 2) to 31.1% (station 4d) and 40.5% (station 3). In patients with tumors in the lower-third, patients with tumors < 4 cm had no pLNMs in stations 2, 3, 4sa, 4sb, 4d, 5, or 6, whereas the incidences of pLNMs in patients with tumors ≥ 4 cm ranged from 5.6% (stations 2, 4sa, or 4sb) to 44.4% (station 6).

### 3.4. Tumor Location Combined with T Stage May Determine pLNM Risk

Among patients with upper-third tumors, T1–T2 patients had no pLNMs in stations 4sa, 4sb, 4d, 5, or 6; T1 patients also had no pLNMs in station 2 (Table 2; Figure S3a,b); T3 patients had no pLNMs in stations 5 and 6; and T4 patients had no pLNMs in station 6 (Table 2; Figure S4a,b). Among the middle-third cases, T1 patients had no pLNMs in stations 2 and 4sb and the incidence of pLNMs in stations 4sa, 5, and 6 was 2.9%; T2 patients had no pLNMs in stations 2, 4sa, 4sb, 5 and 6; T3 patients had no pLNMs in station 2; and the incidences of pLNMs in T4 patients ranged from 14.3% in station 2 to 62.9% in station 3. Among the lower-third cases, there were no pLNMs in any T1–T2 patients except for station 1 (14.3%) in T1 patients and station 6 (50.0%) in T2 patients; T3 patients had no pLNMs in stations 2, 4sa, 4sb, 5, or 6; and the incidences of pLNMs in T4 patients ranged from 7.1% (stations 2, 4sa, and 4sb) to 50.0% (stations 4d and 6). Despite the fact that lymph nodes in stations 4sa and 4sb are required to be dissected in proximal gastrectomy with D1 or D1+ lymphadenectomy, there were no pLNMs in stations 4sa and 4sb among T1–T2 patients with tumors in the upper-third stomach [12].

### 3.5. Identification of Independent Predictive Factors and Models of pLNMs

Univariate analysis identified sex, tumor size, T stage, pathological type, lymphatic invasion, and venous invasion as potentially predictive factors (*p* < 0.05) for entire lymph node metastasis (Table S3). Multivariate analysis (1st) identified pathological type, T stage, lymphatic invasion, and venous invasion as independent predictors for entire lymph node metastasis.

Furthermore, multivariate analysis (2nd) was applied to identify independent predictive factors for perigastric nodal metastasis (Table 3). The predictive model for station 1 pLNM included tumor size, T stage, and lymphatic invasion. The predictive model for station 2 pLNM included sDPD and T stage. The predictive model for station 3 pLNM included T stage and lymphatic invasion. The predictive models for stations 4sa and 4sb included tumor size and T stage, respectively, and the model for 4d included tumor size and sDPD. The predictive model for station 5 pLNM included sDPD and T stage, and the model for station 6 included sDPD and T stage.

### 3.6. Models for pLNM Showed Good Predictive Performance

We assessed the predictive performance of the individual factors and the predictive models for pLNMs, and the models for all pLNMs showed good predictive performance (Table 4; Figure S5).

### 3.7. Indications for Proximal Gastrectomy Could Be Expanded

**Figure 2:**
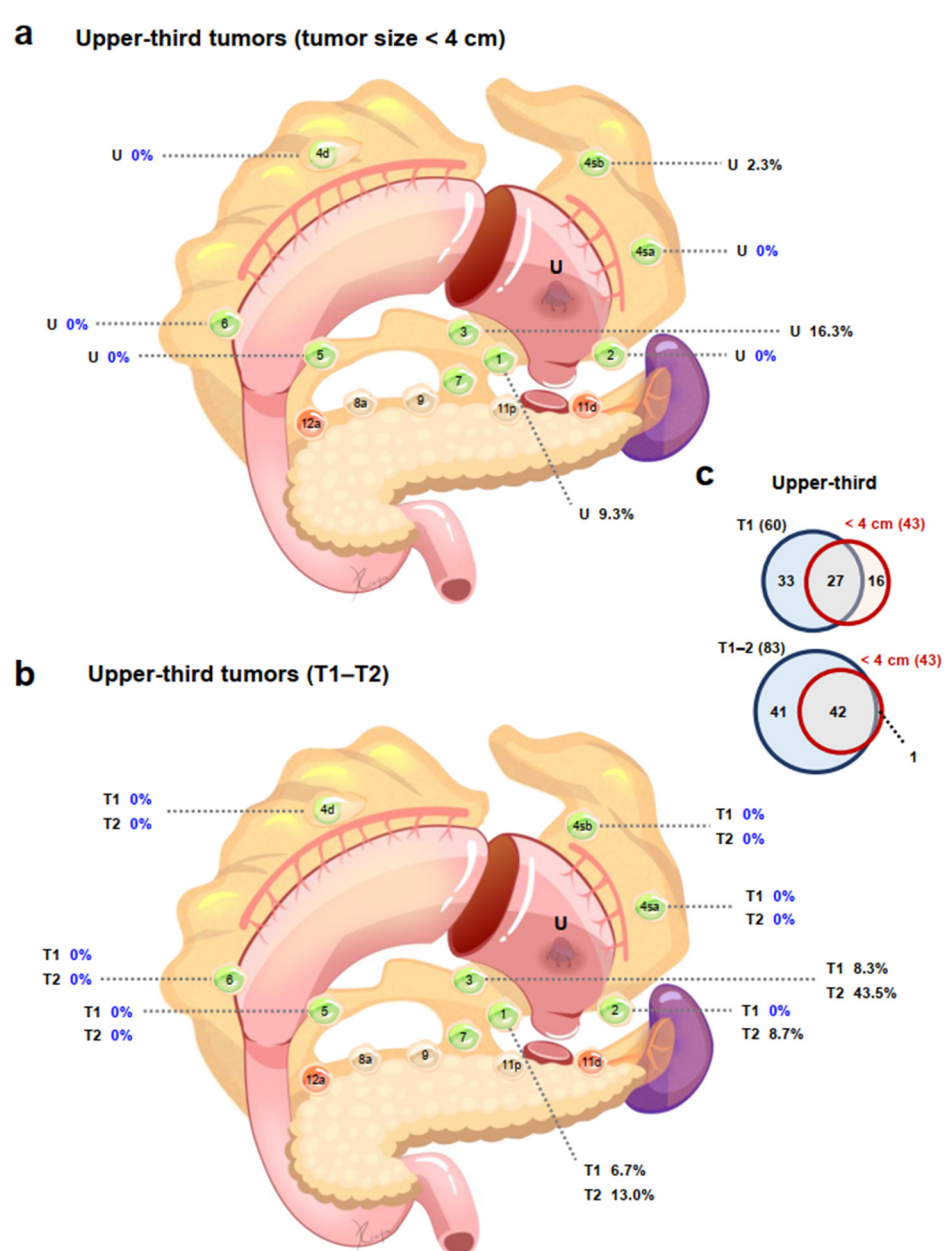
Potential expanded indications for proximal gastrectomy. Upper-third tumors (**a**) <4 cm in diameter and (**b**) T1–T2 stage. (**c**) Numbers of patients in relation to tumor size < 4 cm and T1/T1–T2 stage located in the upper-third of the stomach. U, upper-third tumor.

In the current study, no pLNMs (0/43) were found in stations 4sa, 4d, 5, or 6 in patients with tumors < 4 cm diameter in the upper-third stomach (Figure 2a). In addition, there were no pLNMs in stations 4sa, 4sb, 4d, 5, or 6 in patients with T1 (0/60) or T2 (0/23) upper-third tumors (Figure 2b). Furthermore, tumors with a diameter < 4 cm (42/43) in the upper-third stomach largely overlapped with stage T1–T2 tumors (Figure 2c). Notably, however, 3.8% (2/53) patients in the current study had 4d pLNM.

### 3.8. Indications for PPG May Need Reevaluating

**Figure 3:**
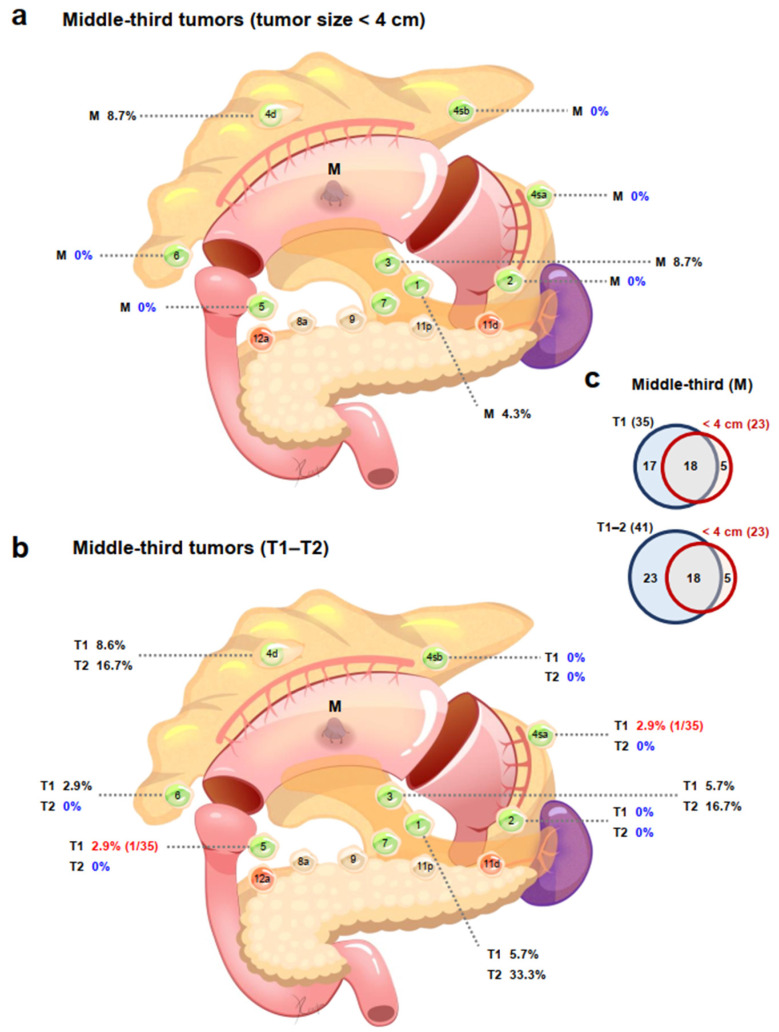
Potential expanded indications for pylorus-preserving gastrectomy. Upper-third tumors (**a**) <4 cm in diameter and (**b**) T1–T2 stage. (**c**) Numbers of patients in relation to tumor size < 4 cm and T1/T1–T2 stage located in the upper-third of the stomach. M, middle-third tumor.

We found no pLNMs in stations 2, 4sa, or 5 in patients with tumors < 4 cm in the middle-third stomach (Figure 3a). For T1 tumors in the middle-third stomach, 2.9% (1/35) patients had 4sa and 5 pLNMs (Figure 3b). Tumors with a diameter < 4 cm (18/23) in the middle-third stomach also largely overlapped with T1–T2 stage tumors (Figure 3c). Moreover, no patients with T2 tumors in the middle-third stomach had pLNMs in stations 2, 4sa, or 5.

## 4. Discussion

In the current study, we explored the signature and prediction of pLNMs in patients with GC after total gastrectomy. We systematically explored the signature of pLNMs in relation to tumor location, tumor size, T stage, and lymphatic invasion, and showed that the combinations of tumor location with tumor size, T stage, and lymphatic invasion may determine the risk of pLNM, and may provide evidence to aid the appropriate selection of surgical procedures. Although various previous methods, including CT [5], DNA methylation markers [6], and the MCP [11], have been used to predict LNMs in patients with GC, the predicted LNMs were not station-specific and further investigations are therefore required. Here, we found that tumor size, T stage, lymphatic invasion, and the novel predictive factor, sDPD, were important predictors of pLNM. The risk of pLNM could also be predicted individually. The signature and prediction of pLNM were important factors informing the choice of gastrectomy. 

Based on the 5th JGCTG, proximal gastrectomy is indicated for cT1N0 proximal tumors when more than half of the distal stomach can be preserved. D1 lymphadenectomy should include stations 1, 2, 3a, 4sa, 4sb, and 7, but dissection of stations 4d, 5, and 6 is not required [12,13]. In the current study, no pLNMs were found in stations 4sa, 4d, 5, or 6 in patients with tumors < 4 cm diameter in the upper-third stomach, and there were no pLNMs in stations 4sa, 4sb, 4d, 5, or 6 in patients with T1–T2 upper-third tumors. Tumors with a diameter < 4 cm in the upper-third stomach largely overlapped with stage T1–T2 tumors. These results suggested that the indications for proximal gastrectomy could be expanded to include T1–T2 tumors and/or tumors < 4 cm diameter in the upper-third stomach, if more than half of the distal stomach can be preserved.

Importantly, our findings were partly consistent with a recent study suggesting that patients with cT1–T2N0/1M0 tumors < 4.1 cm in diameter located in the upper-third stomach could safely undergo proximal gastrectomy [23]. Other studies demonstrated that proximal gastrectomy with exclusion of station 3b lymphadenectomy could be indicated for at least T2 tumors with a diameter < 4 cm in the upper-third stomach [24], while proximal gastrectomy would be the surgery of choice for patients with T2/T3 proximal tumors [9]. Notably however, 3.8% patients in the current study had 4d pLNM. The current evidence thus suggested that T3 tumors in the upper-third stomach may not be indicated for proximal gastrectomy. Proximal gastrectomy without 12a dissection was also reported to be acceptable for upper-third cT2–T4 tumors located in the cardia and/or the fornix, considering the risk of 4d, 5, and 6 pLNM and cancer-positivity in the distal stump [25]. However, the current and previous studies have been retrospective analyses, and further prospective randomized controlled trials comparing proximal gastrectomy with total gastrectomy for cT1–2 tumors and/or tumors < 4 cm in the upper-third are still required to verify these preliminary findings.

PPG can be considered for cT1N0 tumors in the middle-third stomach with a distal tumor border ≥ 4 cm proximal to the pylorus, according to the 5th JGCTG. D1 lymphadenectomy for PPG includes stations 1, 3, 4sb, 4d, 6, and 7, while stations 2, 4sa, and 5 are not routinely dissected. We found no pLNMs in stations 2, 4sa, or 5 in patients with tumors < 4 cm in the middle-third stomach. For T1 tumors in the middle-third stomach, 2.9% patients had 4sa and 5 pLNMs, suggesting that omitting the dissection of stations 4sa and 5 should be re-assessed for patients undergoing PPG. Tumors with a diameter < 4 cm in the middle-third stomach also largely overlapped with T1–T2 stage tumors. Moreover, no patients with T2 tumors in the middle-third stomach had pLNMs in stations 2, 4sa, or 5. These preliminary findings suggested that the indications for PPG may be expanded to include T1–T2N0 tumors and/or tumors < 4 cm diameter in the middle-third stomach, with a distal tumor border ≥ 4 cm proximal to the pylorus. This issue warrants further investigation.

Based on the findings of the current study, both proximal gastrectomy and PPG may be expanded to include patients with stage T1–T2 GC, making it necessary to distinguish between T1–T2 and T3–T4 prior to surgery. CT radiomics, including preoperative arterial-phase and portal-phase contrast-enhanced CT, has been reported to have a potential role in distinguishing T2 and T3–T4 GC [26]. Moreover, endoscopic ultrasound is also an accurate diagnostic device that can be used to preoperatively define the primary tumor depth between T1–T2 and T3–T4 with high sensitivity (0.86) and specificity (0.90), and which might thus be proposed in routine clinical settings prior to surgery [27]. Furthermore, the combination of dynamic contrast-enhanced multislice CT (MSCT) and double contrast-enhanced ultrasound (DCEUS) showed a higher correct-diagnosis rate than MSCT or DCEUS alone in preoperative T staging (T1–T4) [28]. These diagnostic devices may allow physicians to make a correct diagnosis of cT staging, thus allowing the selection of the most appropriate surgical procedures prior to surgery. Importantly, proximal gastrectomy or PPG can be changed to total gastrectomy after T3–T4 confirmation during surgery.

In clinical practice, challenges still exist in terms of determining the most suitable indications for different surgical procedures in patients with GC. First, we should consider the surgical and oncological safety to ensure the best prognosis prior to operation, including the risk of pLNMs, an appropriate type of gastrectomy, adequate extent of lymph node dissection, sufficient resection margin, and suitable reconstruction after gastrectomy. Second, we should also aim to avoid total gastrectomy when possible, considering the importance of the physiological function of the stomach, the effects of gastrectomy on nutritional status, and the quality of life. Third, we should aim to comply with guidelines while questioning their suitability, given that indications may be changed in light of new clinical evidence. Fourth, the findings of this study suggest that some patients may be suitable for partial rather than total gastrectomy, and discussions should be carried out before surgery in these patients. Fifth, if we expand the indications for partial rather than total gastrectomy, careful follow-up should be carried out to detect recurrence or metastases, and postoperative therapy may be necessary in some patients with a risk of recurrence or metastasis. 

The current study had several limitations. First, we included GC patients who received total gastrectomy, and the total number was therefore limited. Second, we analyzed data from patients who underwent total gastrectomy, and the potentially expanded indications suggested by this study should be validated in further prospective, randomized controlled trials comparing partial gastrectomy (e.g., proximal gastrectomy, segmental gastrectomy, and PPG) with total gastrectomy in terms of oncological safety, long-term nutritional outcomes, and the quality of life. Third, station 3 was not subdivided into 3a and 3b in most patients, because only the total number of station 3 lymph nodes (including 3a and 3b) was routinely recorded for a period in our institute. Fourth, we analyzed data from Japanese patients, and the main findings should thus be validated using data from other countries. Fifth, selection bias may exist due to the retrospective nature of this study. The current findings thus need to be interpreted with caution.

## 5. Conclusions

The findings of the current study suggested that the indications for proximal gastrectomy may be expanded to include T1–T2 tumors and/or tumors < 4 cm in diameter in the upper-third stomach, if more than half of the distal stomach can be preserved. The indications for PPG may be expanded to include T1–T2/N0 tumors and/or tumors < 4 cm in diameter in the middle-third stomach, with a distal tumor border ≥ 4 cm proximal to the pylorus. The results also suggest that omitting the dissection of stations 4sa and 5 should be re-assessed for patients undergoing PPG. A new predictive factor, sDPD, showed good predictive performance for pLNMs, especially in stations 4d, 5, and 6.

## Figures and Tables

**Table 1:** Incidence of patients with perigastric lymph node metastasis in relation to tumor location and tumor size.

Station	Upper Third		Middle Third		Lower Third	
	Tumor < 4 cm	Tumor ≥ 4 cm	Tumor < 4 cm	Tumor ≥ 4 cm	Tumor < 4 cm	Tumor ≥ 4 cm
1	9.3% (4/43)	31.6% (42/133)	4.3% (1/23)	28.4% (21/74)	11.1% (1/9)	38.9% (7/18)
2	0% (0/43)	18.8% (25/133)	0% (0/23)	6.8% (5/74)	0% (0/9)	5.6% (1/18)
3	16.3% (7/43)	34.6% (46/133)	8.7% (2/23)	40.5% (30/74)	0% (0/9)	38.9% (7/18)
4sa	0% (0/43)	5.3% (7/133)	0% (0/23)	16.2% (12/74)	0% (0/9)	5.6% (1/18)
4sb	2.3% (1/43)	8.3% (11/133)	0% (0/23)	12.2% (9/74)	0% (0/9)	5.6% (1/18)
4d	0% (0/43)	6.0% (8/133)	8.7% (2/23)	31.1% (23/74)	0% (0/9)	38.9% (7/18)
5	0% (0/43)	0.8% (1/133)	0% (0/23)	10.8% (8/74)	0% (0/9)	11.1% (2/18)
6	0% (0/43)	0% (0/133)	0% (0/23)	16.2% (12/74)	0% (0/9)	44.4% (8/18)

**Table 2:** Incidence of patients with perigastric lymph node metastasis in relation to tumor location and UICC 8th T stage.

Station	Upper Third				Middle Third				Lower Third			
	T1	T2	T3	T4	T1	T2	T3	T4	T1	T2	T3	T4
1	6.7%	13.0%	39.6%	45.0%	5.7%	33.3%	9.5%	45.7%	14.3%	0%	25.0%	42.9%
	(4/60)	(3/23)	(21/53)	(18/40)	(2/35)	(2/6)	(2/21)	(16/35)	(1/7)	(0/2)	(1/4)	(6/14)
2	0%	8.7%	17.0%	35.0%	0%	0%	0%	14.3%	0%	0%	0%	7.1%
	(0/60)	(2/23)	(9/53)	(14/40)	(0/35)	(0/6)	(0/21)	(5/35)	(0/7)	(0/2)	(0/4)	(1/14)
3	8.3%	43.5%	39.6%	42.5%	5.7%	16.7%	33.3%	62.9%	0%	0%	25.0%	42.9%
	(5/60)	(10/23)	(21/53)	(17/40)	(2/35)	(1/6)	(7/21)	(22/35)	(0/7)	(0/2)	(1/4)	(6/14)
4sa	0%	0%	3.8%	12.5%	2.9%	0%	14.3%	22.9%	0%	0%	0%	7.1%
	(0/60)	(0/23)	(2/53)	(5/40)	(1/35)	(0/6)	(3/21)	(8/35)	(0/7)	(0/2)	(0/4)	(1/14)
4sb	0%	0%	5.7%	22.5%	0%	0%	14.3%	17.1%	0%	0%	0%	7.1%
	(0/60)	(0/23)	(3/53)	(9/40)	(0/35)	(0/6)	(3/21)	(6/35)	(0/7)	(0/2)	(0/4)	(1/14)
4d	0%	0%	3.8%	15.0%	8.6%	16.7%	19.0%	48.6%	0%	0%	0%	50.0%
	(0/60)	(0/23)	(2/53)	(6/40)	(3/35)	(1/6)	(4/21)	(17/35)	(0/7)	(0/2)	(0/4)	(7/14)
5	0%	0%	0%	2.5%	2.9%	0%	4.8%	17.1%	0%	0%	0%	14.3%
	(0/60)	(0/23)	(0/53)	(1/40)	(1/35)	(0/6)	(1/21)	(6/35)	(0/7)	(0/2)	(0/4)	(2/14)
6	0%	0%	0%	0%	2.9%	0%	4.8%	28.6%	0%	50.0%	0%	50.0%
	(0/60)	(0/23)	(0/53)	(0/40)	(1/35)	(0/6)	(1/21)	(10/35)	(0/7)	(1/2)	(0/4)	(7/14)

UICC, Union for International Cancer Control.

**Table 3:** Identification of predictors of perigastric lymph node metastases by multivariate analyses.

Station	Multivariate Analysis (2nd)	Tumor Size (Continuous)	sDPD (Continuous)	Pathological Type	UICC 8th T Stage	Lymphatic Invasion	Venous Invasion
1	OR	1.010	1.025	1.254	1.406	2.575	1.945
	(95% CI)	(1.001–1.018)	(0.962–1.092)	(0.868–1.810)	(1.030–1.918)	(1.172–5.653)	(0.769–4.916)
	*p* value	0.021	0.445	0.228	0.032	0.018	0.160
2	OR	1.004	1.121	1.221	2.478	2.523	3.283
	(95% CI)	(0.993–1.014)	(1.024–1.228)	(0.716–2.083)	(1.417–4.333)	(0.673–9.455)	(0.387–27.85)
	*p* value	0.485	0.014	0.463	0.001	0.170	0.276
3	OR	1.007	0.997	1.404	1.416	2.110	1.493
	(95% CI)	(0.999–1.015)	(0.940–1.058)	(0.999–1.972)	(1.059–1.893)	(1.050–4.242)	(0.665–3.349)
	*p* value	0.080	0.925	0.051	0.019	0.036	0.331
4sa	OR	1.019	1.032	1.109	1.739	2.647	0.728
	(95% CI)	(1.008–1.030)	(0.923–1.154)	(0.558–2.204)	(0.913–3.314)	(0.501–13.97)	(0.149–3.555)
	*p* value	0.001	0.577	0.768	0.092	0.252	0.695
4sb	OR	1.006	1.074	1.273	2.468	5.283	1.735
	(95% CI)	(0.994–1.017)	(0.970–1.190)	(0.672–2.410)	(1.265–4.816)	(0.645–43.29)	(0.194–15.48)
	*p* value	0.319	0.170	0.458	0.008	0.121	0.622
4d	OR	1.009	0.882	1.214	1.480	2.414	4.543
	(95% CI)	(1.000–1.018)	(0.808–0.962)	(0.720–2.048)	(0.972–2.251)	(0.736–7.925)	(0.884–23.34)
	*p* value	0.045	0.005	0.466	0.067	0.146	0.070
5	OR	0.983	0.761	1.199	2.454	0.964	1.263
	(95% CI)	(0.963–1.003)	(0.633–0.914)	(0.489–2.940)	(1.050–5.739)	(0.146–6.359)	(0.102–15.65)
	*p* value	0.097	0.004	0.692	0.038	0.969	0.856
6	OR	0.993	0.676	0.885	1.782	1.860	9.466
	(95% CI)	(0.981–1.006)	(0.567–0.804)	(0.431–1.817)	(1.011–3.142)	(0.405–8.533)	(0.575–155.8)
	*p* value	0.280	<0.001	0.739	0.046	0.425	0.116

Logistic regression analyses were performed for multivariate analyses. CI, confidence interval; sDPD, the shortest distance from the pylorus ring to distal edge of the tumor; OR, odds ratio; UICC, Union for International Cancer Control.

**Table 4:** Predictive performance of predictors for perigastric lymph node metastases.

Perigastric Lymph Node Metastasis (pLNM) Prediction	AUC (95% CI)	AIC
Station 1 pLNM		
Tumor size	0.706 (0.650–0.757)	320
UICC 8th T stage	0.727 (0.673–0.777)	307
Lymphatic invasion	0.682 (0.626–0.734)	309
Model: Tumor size + UICC 8th T stage + lymphatic invasion	0.774 (0.723–0.820)	297
Station 2 pLNM		
sDPD	0.553 (0.495–0.610)	203
UICC 8th T stage	0.775 (0.724–0.821)	174
Model: sDPD + UICC 8th T stage	0.811 (0.763–0.854)	171
Station 3 pLNM		
UICC 8th T stage	0.719 (0.665–0.770)	322
Lymphatic invasion	0.667 (0.611–0.720)	342
Model: UICC 8th T stage + lymphatic invasion	0.742 (0.689–0.791)	317
Station 4sa pLNM		
Tumor size	0.813 (0.765–0.856)	126
Station 4sb pLNM		
UICC 8th T stage	0.794 (0.744–0.839)	136
Station 4d pLNM		
Tumor size	0.757 (0.704–0.804)	213
sDPD	0.725 (0.671–0.775)	218
Model: Tumor size + sDPD	0.819 (0.771–0.861)	206
Station 5 pLNM		
sDPD	0.741 (0.687–0.790)	85
UICC 8th T stage	0.752 (0.699–0.800)	90
Model: sDPD + UICC 8th T stage	0.791 (0.740–0.835)	85
Station 6 pLNM		
sDPD	0.873 (0.829–0.908)	112
UICC 8th T stage	0.804 (0.755–0.848)	127
Model: sDPD + UICC 8th T stage	0.909 (0.871–0.939)	103

AIC, Akaike information criterion; AUC, area under the receiver operating characteristic curve; pLNM, perigastric lymph node metastasis; sDPD, shortest distance from pylorus ring to distal edge of the tumor; UICC, Union for International Cancer Control. A higher AUC indicates better model discrimination, while a lower AIC indicates superior model fitting.

## Data Availability

The data presented in this study are available on request from the corresponding author. The data are not publicly available due to patient privacy and the General Data Protection Regulations.

## Supplementary Materials

The following supporting information can be downloaded at: https://www.mdpi.com/article/10.3390/cancers14143409/s1, Figure S1: Study flow diagram, Figure S2: Incidence of patients with perigastric lymph node metastasis. Upper-, middle-, and lower-third tumors (a) < 4 cm and (b) ≥ 4 cm in diameter, Figure S3: Incidence of patients with perigastric lymph node metastasis. Upper-, middle-, and lower-third tumors of (a) T1 stage and (b) T2 stage, Figure S4: Incidence of patients with perigastric lymph node metastasis. (a) Upper-, middle-, and lower-third tumors of (a) T3 stage and (b) T4 stage, Figure S5: Area under the receiver operating characteristic curves for factors predicting perigastric lymph node metastases in stations 1, 2, 3, 4sa, 4sb, 4d, 5, and 6. sDPD, the shortest distance from the pylorus ring to distal edge of the tumor, Table S1: Baseline characteristics in relation to tumor location, Table S2: Perigastric lymph node metastasis rate in relation to tumor location, Table S3: Univariate and multivariate analyses of predictive factors for entire lymph node metastasis.
