# Supplementary material for: Signature and Prediction of Perigastric Lymph Node Metastasis in Patients with Gastric Cancer and Total Gastrectomy: Is Total Gastrectomy Always Necessary?
Source: Cancers (Basel). 2022 Jul 13;14(14):3409. doi: 10.3390/cancers14143409 (PMC9319199; doi:10.3390/cancers14143409)
Supplement: Supplementary file 1 [file cancers-14-03409-s001.zip › Supplementary Figures.pdf]

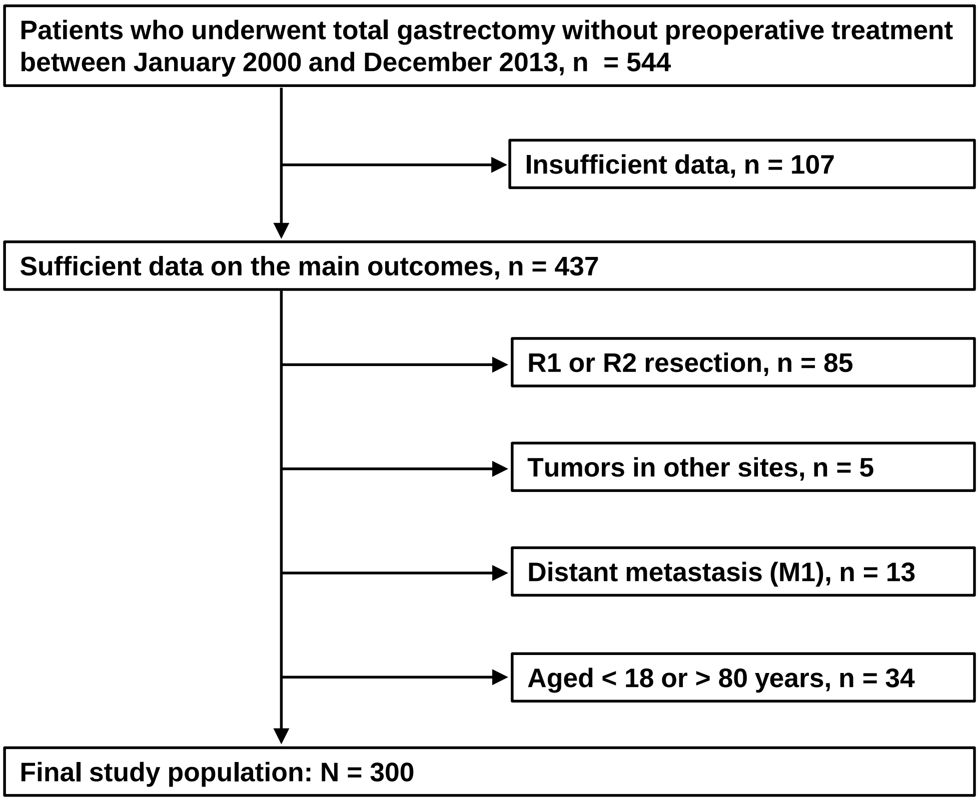

Figure S1. Study flow diagram

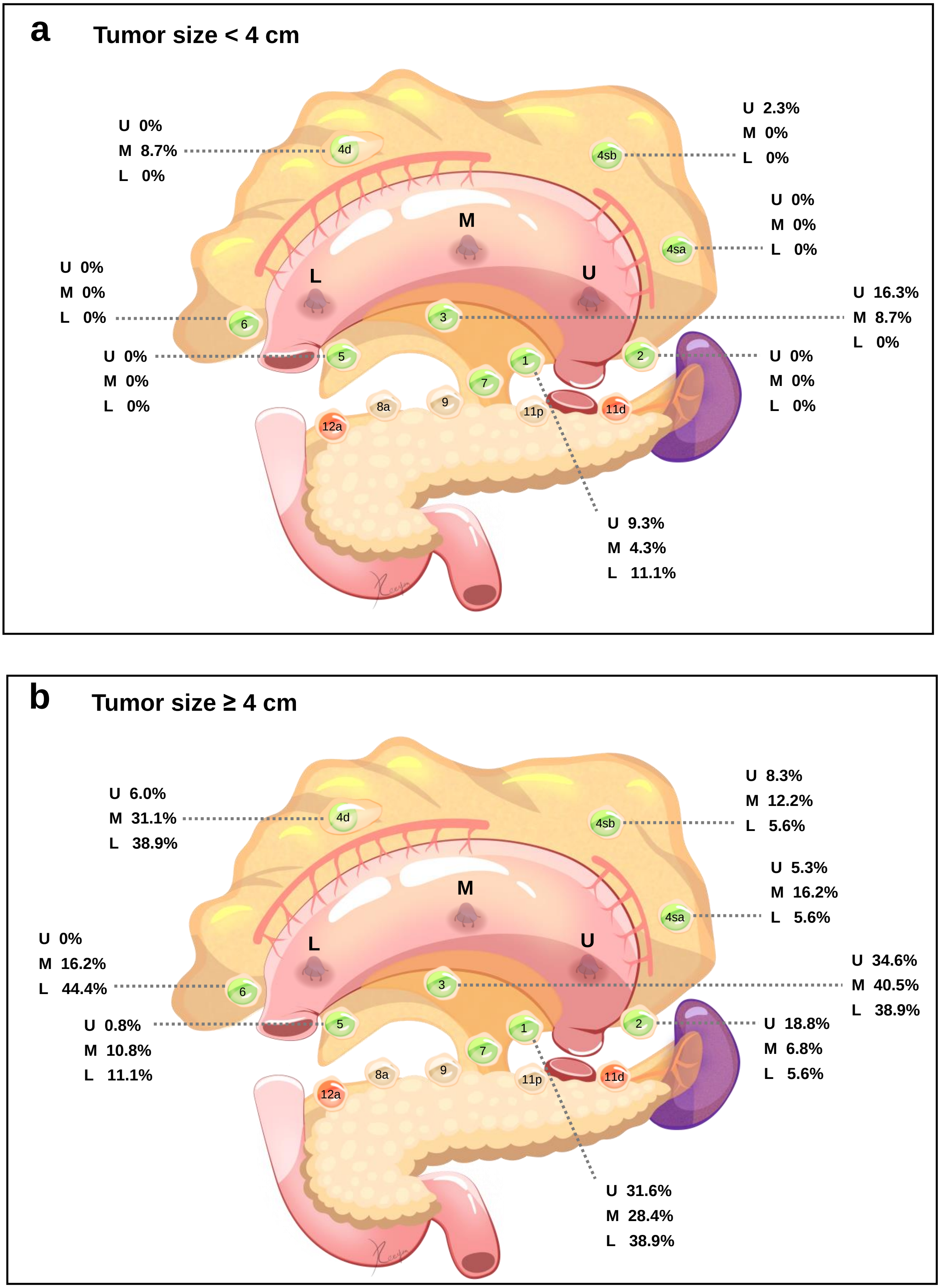

Figure S2. Incidence of patients with perigastric lymph node metastasis. Upper-, middle-, and lower-third tumors (a) < 4 cm and (b) ≥ 4 cm in diameter.

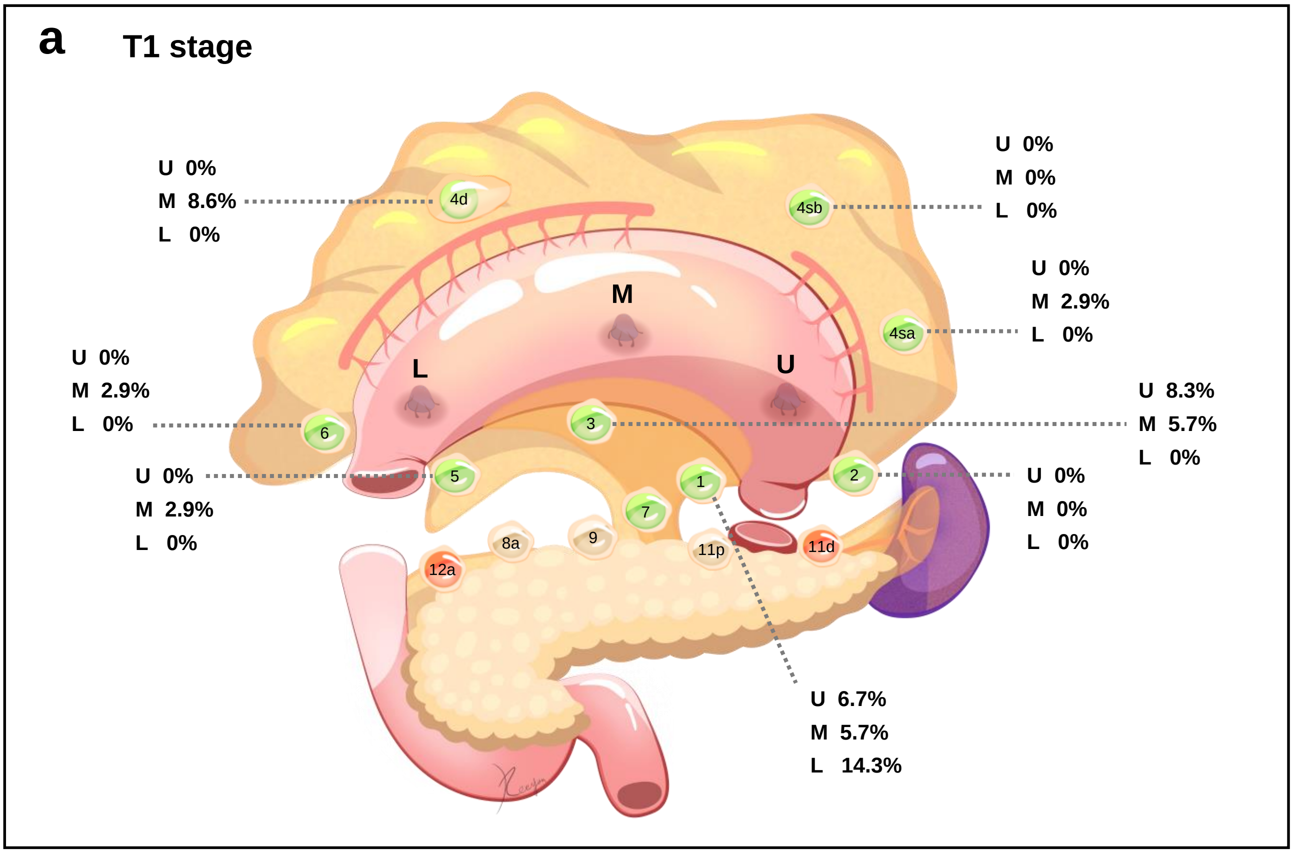

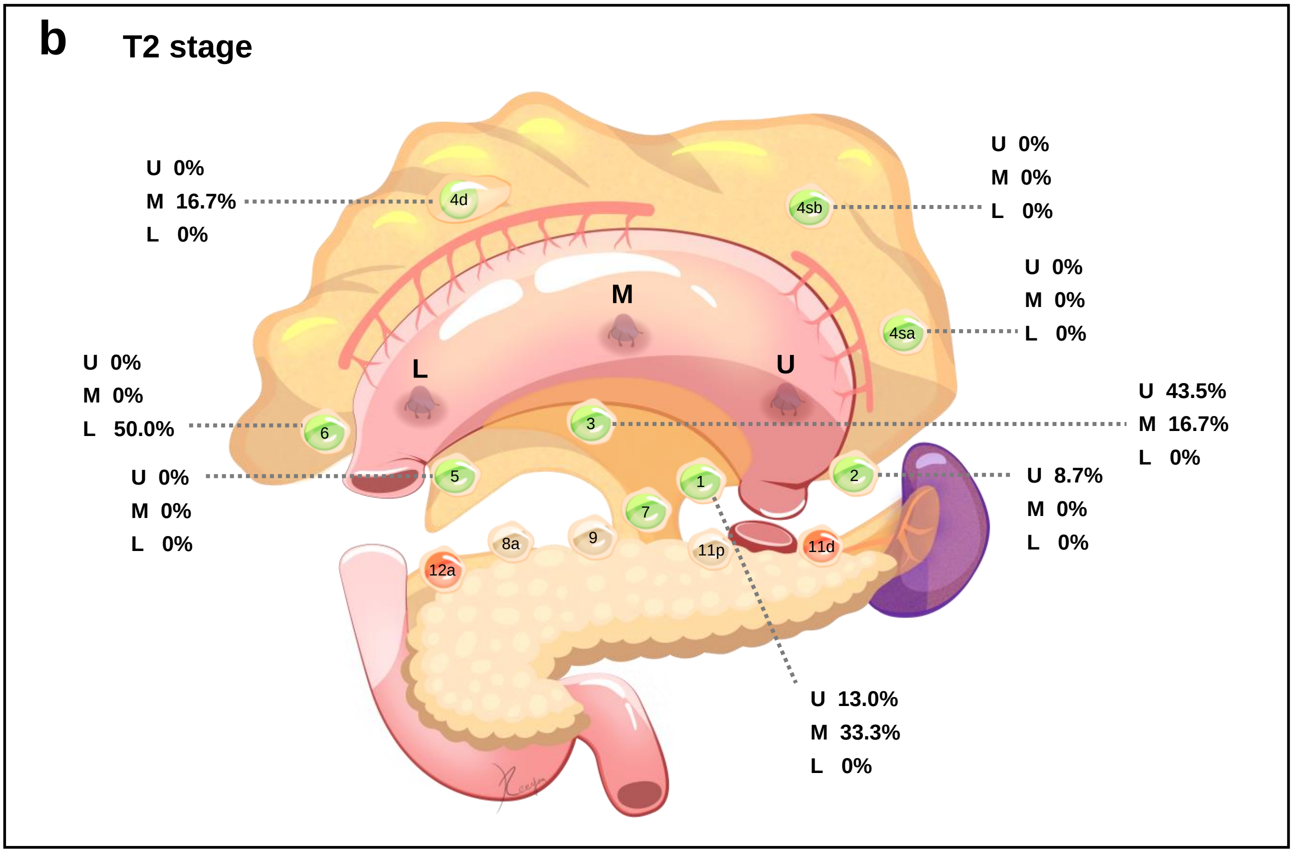

Figure S3. Incidence of patients with perigastric lymph node metastasis. Upper-, middle-, and lower-third tumors of (a) T1 stage and (b) T2 stage.

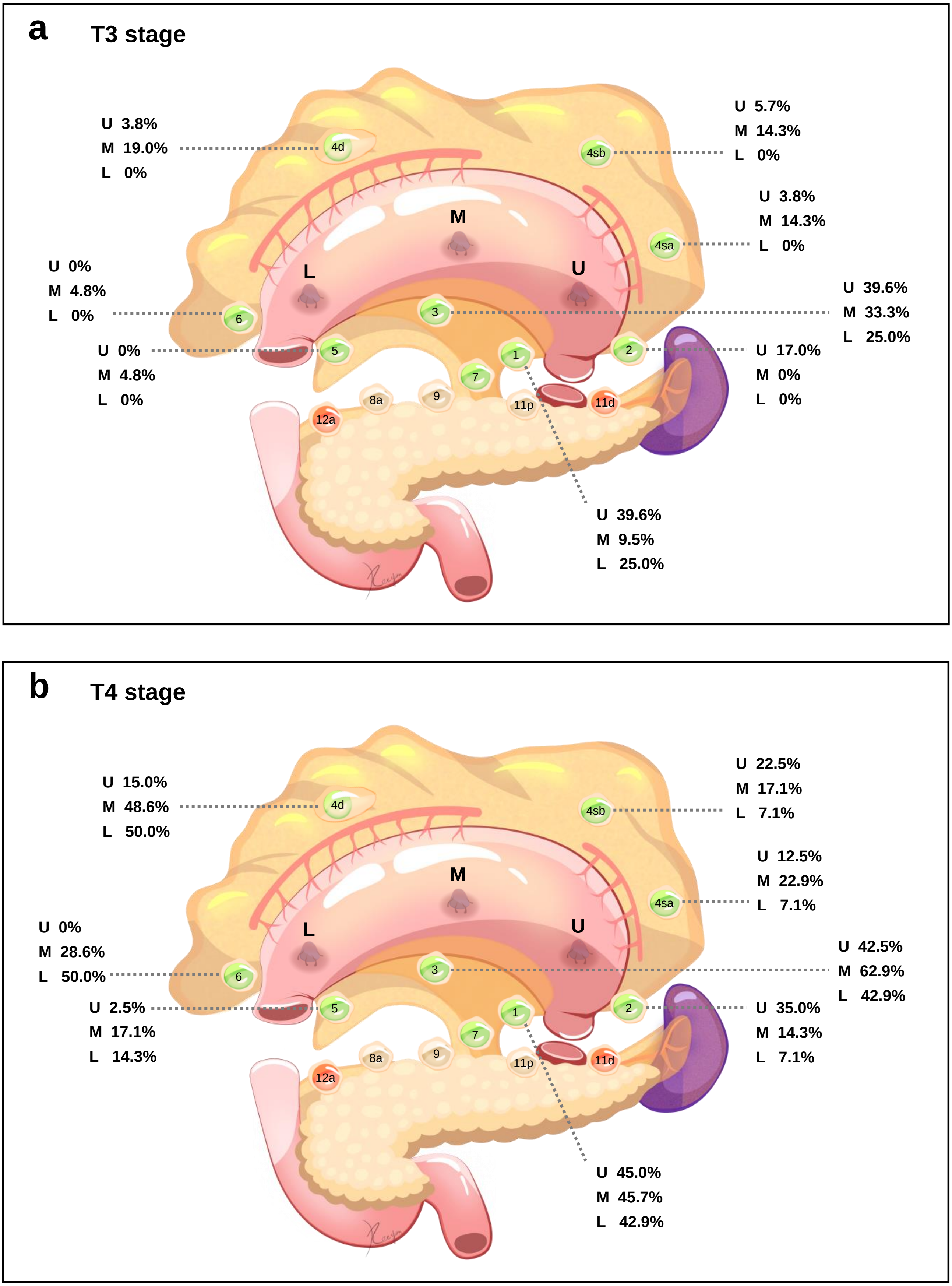

Figure S4. Incidence of patients with perigastric lymph node metastasis. (a) Upper-, middle-, and lower-third tumors of (a) T3 stage and (b) T4 stage.

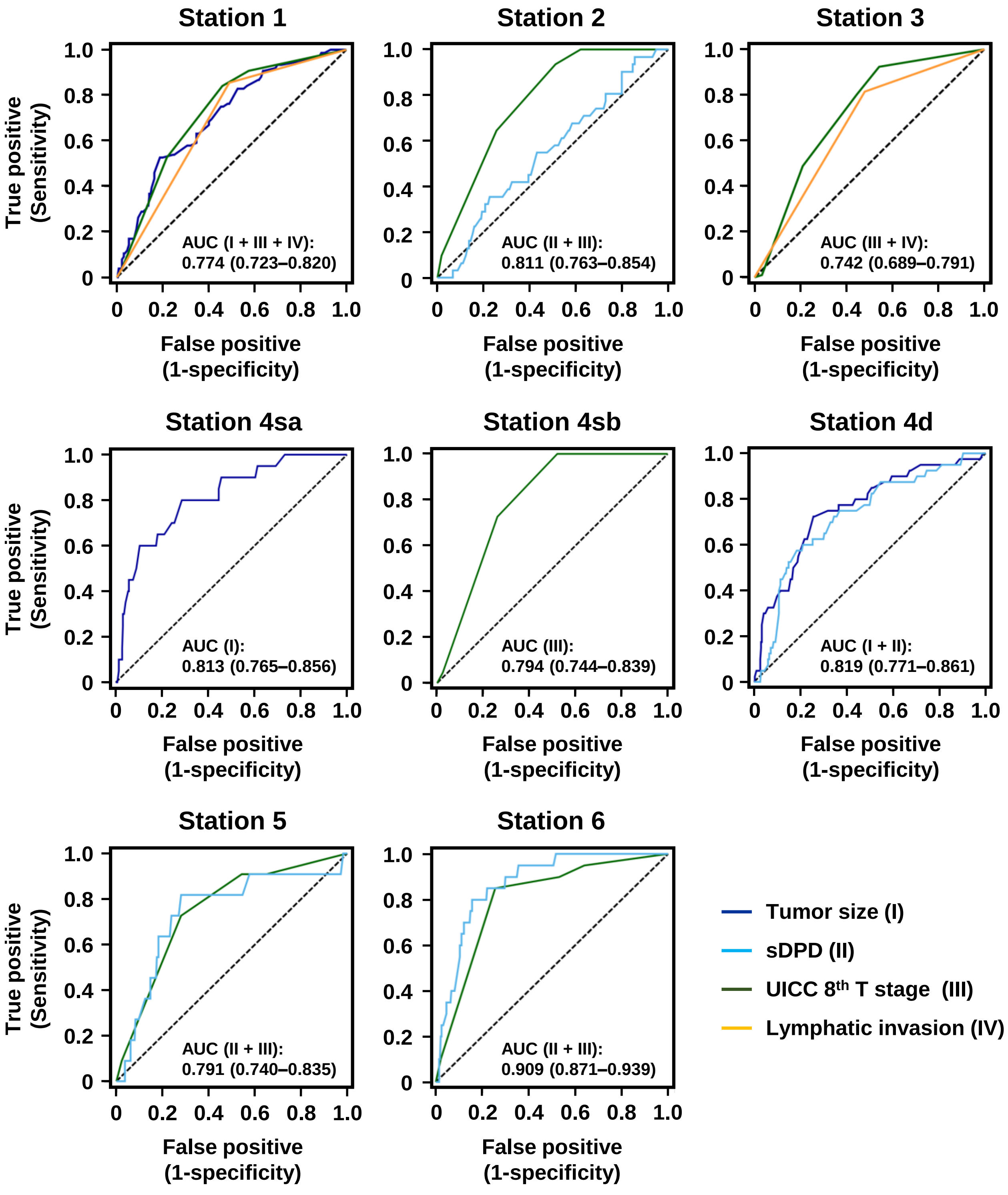

Figure S5. Area under the receiver operating characteristic curves for factors predicting perigastric lymph node metastases in stations 1, 2, 3, 4sa, 4sb, 4d, 5, and 6. sDPD, the shortest distance from the pylorus ring to distal edge of the tumor
