# Supplementary material for: Signature and Prediction of Perigastric Lymph Node Metastasis in Patients with Gastric Cancer and Total Gastrectomy: Is Total Gastrectomy Always Necessary?
Source: Cancers (Basel). 2022 Jul 13;14(14):3409. doi: 10.3390/cancers14143409 (PMC9319199; doi:10.3390/cancers14143409)
Supplement: Supplementary file 1 [file cancers-14-03409-s001.zip › Supplementary Tables.pdf]

Table S1. Baseline characteristics in relation to tumor location.

Variables	Upper third	Middle third	Lower third	Overall
Sex (%)				
Male	137 (77.8)	73 (75.3)	24 (88.9)	234 (78.0)
Female	39 (22.2)	24 (24.7)	3 (11.1)	66 (22.0)
Age, year (%)				
Median (range)	67.5 (28–80)	64 (35–79)	65 (49–80)	66.5 (28–80)
< 65	69 (39.2)	50 (51.5)	13 (48.1)	132 (44.0)
≥ 65	107 (60.8)	47 (48.5)	14 (51.9)	168 (56.0)
Tumor size, cm (%)				
Median (range)	5.2 (0.6–21.0)	6.5 (1.2–20.5)	6.2 (1.1–17.0)	5.5 (0.6–21.0)
< 4.0	43 (24.4)	23 (23.7)	9 (33.3)	75 (25.0)
≥ 4.0	133 (75.6)	74 (76.3)	18 (66.7)	225 (75.0)
Cross section (%)				
Lesser curvature	75 (42.6)	46 (47.4)	14 (51.9)	135 (45.0)
Great curvature	10 (5.7)	10 (10.3)	0 (0)	20 (6.7)
Anterior wall	28 (15.9)	12 (12.4)	2 (7.4)	42 (14.0)
Posterior wall	54 (30.7)	14 (14.4)	7 (25.9)	75 (25.0)
Circle	9 (5.1)	15 (15.5)	4 (14.8)	28 (9.3)
UICC 8th T stage (%)				
T1 (M/SM)	60 (34.1)	35 (36.1)	7 (25.9)	102 (34.0)
T2 (MP)	23 (13.1)	6 (6.2)	2 (7.4)	31 (10.3)
T3 (SS)	53 (30.1)	21 (21.6)	4 (14.8)	78 (26.0)
T4a (S)	37 (21.0)	31 (32.0)	13 (48.1)	81 (27.0)
T4b (SI)	3 (1.7)	4 (4.1)	1 (3.7)	8 (2.7)
UICC 8th N stage (%)				
N0	80 (45.5)	42 (43.3)	11 (40.7)	133 (44.3)
N1	37 (21.0)	17 (17.5)	4 (14.8)	58 (19.3)
N2	22 (12.5)	16 (16.5)	5 (18.5)	43 (14.3)
N3a	24 (13.6)	10 (10.3)	5 (18.5)	39 (13.0)
N3b	13 (7.4)	12 (12.4)	2 (7.4)	27 (9.0)
Pathological type (%)				
Well differentiated	42 (23.9)	24 (24.7)	13 (48.1)	79 (26.3)
Poorly differentiated	34 (19.3)	29 (29.9)	3 (11.1)	66 (22.0)
Mixed type	94 (53.4)	43 (44.3)	11 (40.7)	148 (49.3)
Others	6 (3.4)	1 (1.0)	0 (0)	7 (2.3)
Retrieved lymph nodes (%)				
Median (range)	49 (14–129)	49 (11–132)	47 (14–98)	48 (11–132)
Adequate (n ≥ 16)	174 (98.9)	94 (96.9)	26 (96.3)	294 (98.0)
Inadequate (n < 16)	2 (1.1)	3 (3.1)	1 (3.7)	6 (2.0)
Positive lymph nodes				
Median (range)	1 (0–29)	1 (0–83)	2 (0–22)	1 (0–83)

Lymphatic invasion (%)				
No	75 (42.6)	40 (41.2)	10 (37.0)	125 (41.7)
Yes	101 (57.4)	57 (58.8)	17 (63.0)	175 (58.3)
Venous invasion (%)				
No	56 (31.8)	34 (35.1)	6 (22.2)	96 (32.0)
Yes	120 (68.2)	63 (64.9)	21 (77.8)	204 (68.0)
Adjuvant chemotherapy (%)				
No	95 (54.0)	47 (48.5)	13 (48.1)	155 (51.7)
Yes	81 (46.0)	50 (51.5)	14 (51.9)	145 (48.3)
sDPD, cm				
Median (range)	12.0 (0–21.0)	8.0 (0–19.0)	3.0 (0–9.5)	10.0 (0–21.0)

UICC, Union for International Cancer Control; sDPD, the shortest distance from the pylorus ring to distal edge of the tumor.

Table S2. Perigastric lymph node metastasis rate in relation to tumor location.

Station	Upper third Total number for 176 patients			Middle third Total number for 97 patients			Lower third Total number for 27 patients		
	Total LNs	Metastasis LNs	Metastasis rate, %	Total LNs	Metastasis LNs	Metastasis rate, %	Total LNs	Metastasis LNs	Metastasis rate, %
1	1000	106	10.6%	489	52	10.6%	133	16	12.0%
2	568	58	10.2%	241	15	6.2%	69	2	2.9%
3	1277	144	11.3%	673	130	19.3%	134	25	18.7%
4sa	335	16	4.8%	270	50	18.5%	46	3	6.5%
4sb	489	27	5.5%	273	24	8.8%	60	5	8.3%
4d	1324	18	1.4%	800	91	11.4%	155	17	11.0%
5	121	1	0.8%	54	8	9.3%	15	2	13.3%
6	764	0	0%	464	41	8.8%	134	15	11.2%

LNs, lymph nodes; n, number of patients.

Table S3. Univariate and multivariate analyses of predictive factors for entire lymph node metastasis.

Variables	Univariate analysis			Multivariate analysis (1st)		
	**OR**	**95% CI**	***P* value**	**OR**	**95% CI**	***P* value**
Sex (male / female)	1.967	1.104–3.506	0.022	1.927	0.905–4.103	0.086
Age (continuous variable)	0.994	0.972–1.016	0.592	–	–	–
Tumor size (continuous variable)	1.021	1.013–1.029	<0.001	1.007	0.998–1.016	0.105
Tumor location (upper / middle / lower)	1.097	0.774–1.555	0.602	–	–	–
Cross section (Les / Gre / Ant / Post / Cir)	1.009	0.866–1.176	0.906	–	–	–
sDPD (continuous variable)	0.984	0.941–1.029	0.478	–	–	–
Pathological type (well / poorly / mixed / others)	1.767	1.349–2.315	<0.001	1.492	1.072–2.076	0.018
UICC 8th T stage (T1 / T2 / T3 / T4a / T4b)	2.707	2.146–3.413	<0.001	1.726	1.290–2.310	<0.001
Retrieved lymph nodes (adequate / inadequate)	0.793	0.157–3.993	0.778	–	–	–
Lymphatic invasion (no / yes)	7.894	4.691–13.28	<0.001	2.439	1.284–4.634	0.006
Venous invasion (no / yes)	8.267	4.703–14.53	<0.001	2.900	1.399–6.011	0.004

Logistic regression analyses were performed for univariate and multivariate analyses. CI, confidence interval; n, number of patients; sDPD, the shortest distance from the pylorus ring to distal edge of the tumor; OR, odds ratio; OS, overall survival; UICC, Union for International Cancer Control; Les, lesser curvature; Gre, great curvature; Ant, anterior wall; Post, posterior wall; Cir, circle. Variables with *P* values < 0.05 in univariate analysis were applied in multivariate analysis.
